# Cryptanalysis of a New Chaotic Image Encryption Technique Based on Multiple Discrete Dynamical Maps

**DOI:** 10.3390/e23121581

**Published:** 2021-11-26

**Authors:** Haiju Fan, Chenjiu Zhang, Heng Lu, Ming Li, Yanfang Liu

**Affiliations:** 1 College of Computer and Information Engineering, Henan Normal University, Xinxiang 453007, China; 121064@htu.edu.cn (H.F.); untoldsea@163.com (H.L.); liming@htu.edu.cn (M.L.); 121101@htu.edu.cn (Y.L.); 2 Engineering Lab of Intelligence Business & Internet of Things, Xinxiang 453007, China; 3 Big Data Engineering Lab of Teaching Resources & Assessment of Education Quality, Xinxiang 453007, China; 4 Key Laboratory of Artificial Intelligence and Personalized Learning in Education of Henan Province, Xinxiang 453007, China

**Keywords:** image encryption, multiple dynamic maps, permutation–diffusion structure, cryptanalysis, chosen-plaintext attack

## Abstract

Recently, a new chaotic image encryption technique was proposed based on multiple discrete dynamic maps. The authors claim that the scheme can provide excellent privacy for traditional digital images. However, in order to minimize the computational cost, the encryption scheme adopts one-round encryption and a traditional permutation–diffusion structure. Through cryptanalysis, there is no strong correlation between the key and the plain image, which leads to the collapse of cryptosystem. Based on this, two methods of chosen-plaintext attacks are proposed in this paper. The two methods require 3 pairs and 258 pairs of plain and cipher images, respectively, to break the original encryption system. The simulation results show the effectiveness of the two schemes.

## 1. Introduction

In this era of big data, information security has always been a sensitive topic [1]. Image files as one of the most typical multimedia information will inevitably pass through unstable and unsafe channels, and probably resulting in information leakage. Criminals intercept important information by certain technical means to change and transmit it at will, causing a series of information security problems [2]. Over the years, a large number of researchers have proposed a variety of image encryption ideas and strived to solve the security of image transmission. Among them, chaos-based methods are more suitable for image encryption than DES [3] and AES [4] because of the characteristics of pseudo-randomness and sensitivity. Therefore, chaos-based image encryption schemes have sprung up owing to the researchers who are investing a lot of time and effort in this field [5,6,7,8,9,10,11,12].

So far, many chaos-based image encryption schemes have been based on the permutation–diffusion structure proposed by Fridrich [13]. However, some encryption schemes [14,15,16,17,18] are insecure, which have been cracked by some researchers with known/chosen-ciphertext attacks or known/chosen-plaintext attacks [19,20,21,22,23]. Hu et al. in [17] found security vulnerabilities in the scheme [24] and carried out cryptanalysis and improvement. In the improved scheme, different secret keys were proposed to eliminate vulnerabilities in two rounds of diffusion. However, this scheme was still cracked by Li et al. in [25], who found a linear relationship of diffusion and used chosen-plaintext attacks. These broken encryption schemes are problematic because the key is fixed—it cannot be adapted to different plain images. At this point, the plain/cipher images can be constructed. The generated plain/cipher is then used to infer the key. To overcome this shortcoming, Hsiao et al. in [26] proposed a color image encryption scheme that uses the sum of plain image pixels to enhance the secret key space. The sum of its pixels (Mod 256) is set as the basic parameter of chaotic sequence. Since the sum of its pixels (Mod 256) has 256 possibilities, it can be exhaustively listed and calculated for correlation analysis. Having found the most relevant image among 256 plain/cipher images, this scheme was cracked by Fan et al. in [27] with a chosen-plaintext attack. However, the broken encryption scheme exposed some features of the plain image due to the low-level key space and square image limitations. The attacker uses the number of key matrices and the range of pixel values in encryption to successfully crack the cryptosystem through correlation analysis. In [28], an image encryption algorithm based on improved Henon mapping is proposed to encrypt images through shift transformation and diffusion. This scheme was effectively cracked by Zhou et al. in [29] using 257 plaintext images, and if boundary pixels were not taken into account, two plaintext images were required to achieve effectively attack. Mondal et al. in [30] proposed a new 2D chaotic mapping. The encryption scheme first scrambles the pixel position and then changes the pixel position by diffusion. Li et al. in [31] found three security vulnerabilities in this scheme, and successfully cracked them by using a chosen-plaintext attack. Although these schemes involve multiple rounds of encryption with a high level of time complexity, they are all based on the permutation–diffusion structure, and the key is independent of the plain image. Therefore, the attacker can first eliminate the diffusion effect of the cipher image and observe the different characteristics leaked in the encryption stage, and then verify possible solutions to break the stages of multiple rounds of permutation and diffusion.

Recently, Majid in [32] proposed a new chaotic image encryption technique based on multiple discrete dynamical maps. It uses three chaotic maps to generate random sequences, and in order to save time, it only uses one round of encryption to achieve confusion and diffusion. The scheme can be divided into two parts: pixel permutation and pixel diffusion. This scheme involves one round of encryption based on a permutation–diffusion structure, which has been analyzed and studied by many researchers [33,34]. We conducted cryptanalysis on the encryption scheme and then proposed related attack methods to crack it. In this paper, we have used two methods of chosen-plaintext attack, both of which can destroy diffusion and permutation transformation, and successfully attacked the original encryption scheme. The rest of the paper is organized as follows: Section 2 summarizes Majid’s scheme [30]; Section 3 proposes two kinds of chosen-plaintext attacks and carries out simulation experiments; the final section simply summarizes the whole paper.

## 2. Overview of Majid’s Encryption Scheme

Majid et al. designed an original image encryption scheme, which used one-round encryption to provide better confidentiality. This encryption scheme is divided into five main steps: image sub-blocking, permutation within blocks, permutation between blocks, the first diffusion, and the second diffusion.

### 2.1. Chaotic Maps of the Original Cipher Scheme

A two-dimensional Henon chaotic map has good chaotic behavior, and its mathematical expression is presented as follows:

(1) $$x_{n+1}=1-ax_{n}+y_{n},$$

(2) $$y_{n+1}=bx_{n},$$

where *x*_0_ and *y*_0_ are the initial parameters of the chaotic map, and *x*_0_ = 1.61001, *y*_0_ = 2.9996, *a* = 1.7085, *b* = 0.32032.

A circle chaotic map has good chaotic behavior, and its mathematical expression is as follows:

(3) $$\theta_{n+1}=\mathrm{mod}(\theta_{n}+\Omega -\frac{K}{2\pi }\sin(2\pi (\theta_{n})),1),$$

where *θ*_0_ = 0.4, Ω = 0.4, *K* is a constant.

A Duffing chaotic map also has good chaotic behavior. Its mathematical expression is as follows:

(4) $$x_{n+1}=y_{n},$$

(5) $$y_{n+1}=-bx_{n}+ay_{n}-y_{n}{}^{3},$$

where *x*_0_ = −1.5, *y*_0_ = 1.5, *a* = 2.738, *b* = 0.1534.

### 2.2. Majid’s Encryption Scheme

The specific steps are as follows:

**Step 1**  Input a 256 × 256 × 3 plain image, divide it into three channels, and then separate the single-channel image into 32 × 32 blocks.
**Step 2**  Generate a chaotic sequence using a two-dimensional Henon chaotic map and perform pixel permutation within blocks of each channel in order.
**Step 3**  Perform random permutation between blocks of each channel orderly.
**Step 4**  Use a circle chaotic map to execute the first diffusion for each channel.
**Step 5**  Use a Duffing chaotic map to execute the second diffusion for each channel.

## 3. Cryptanalysis

This section mainly introduces the preparation work and two schemes to eliminate the diffusion effect and obtain equivalent displacement mapping. The feasibility of the two schemes is verified by simulation experiments.

### 3.1. Preparations

**Proposition** **1.** *For the encryption scheme of the permutation–diffusion structure, using the all-zero image can break the diffusion transform to obtain the equivalent diffusion matrix.*

**Proof.**  Assuming that the plain image of 256 × 256 × 3 is *P_i_*, the permutation transformation expression is defined as:

(6) $$S=F_{i}(P_{i}),$$

where *F_i_*(.) is the permutation transform of the image, *S* is the permutation-only image of plain image *P_i_*. During the permutation transformation, the pixel value *P_i_*(*i*, *j*) in the plain image only corresponds to the pixel value *S*(*i*, *j*) in the permutation-only image of row *i* and column *j*.

The expression of image diffusion transformation is as follows:

(7) $$D=S⊕D_{i},$$

where ⊕ is the XOR operation, *D_i_* is the diffusion matrix, and *D* is the image after diffusion transformation. Finally, the cipher image *C_i_* corresponding to the plain image *P_i_* is obtained as shown in Equation (8).

(8) $$C_{i}=F_{i}(P_{i})⊕D_{i}=S⊕D_{i}.$$

The permutation phase will not change the original pixel value of the image but simply changes the pixel position of the previous image. A plain image of all zero pixels is still with all zero pixels after permutation. Then, one will obtain the cipher image *C*_0_ from the all-zero image *P*_0_ after the diffusion step.

(9) $$C_{0}=F_{i}(P_{0})⊕D_{i}=P_{0}⊕D_{i}=D_{i}.$$

It can be seen that after a round of permutation and diffusion of all-zero image *P*_0_, the cipher image *C*_0_ obtained at this time is exactly equal to diffusion matrix *D_i_*. Then, the permutation-only image *S* can be obtained by XOR operation in Equation (10).

(10) $$C_{i}⊕C_{0}=S⊕D_{i}⊕D_{i}=S.$$

□

**Proposition** **2.** *Two diffusions on a permutation-only image S can be linearly transformed into one diffusion.*

**Proof.**  (11) $$C_{i}(i,j)=S(i,j)⊕D_{1}(i,j)⊕D_{2}(i,j),$$

where *D*_1_ is the first diffusion matrix and *D*_2_ is the second diffusion matrix.

At this time, according to the reversible operation of XOR, the XOR result of the next two matrices is calculated firstly as follows:

(12) $$D_{i}(i,j)=D_{1}(i,j)⊕D_{2}(i,j).$$

Finally, the pixel value of *D_i_*(*i*, *j*) is obtained and XOR operation is performed with the pixel value of *S*(*i*, *j*).

(13) $$C_{i}(i,j)=S(i,j)⊕D_{i}(i,j).$$

Then,

(14) $$C_{i}=S⊕D_{i}.$$

□

**Proposition** **3.** *Suppose a plain image is denoted by P*_1_*, and P*_2_* is obtained by changing n pixels of image P*_1_*. Then, P*_1_* and P*_2_* are encrypted to obtain the cipher images C*_1_* and C*_2_*. So, C*_1_* and C*_2_* will have n different pixel values. The pixel value of each position in the plain image is one-to-one mapped to the pixel value of a certain position in the cipher image, that is, there is a mapping relationship between them.*

**Proof.**  The encryption process for plain image *P*_1_ is as follows:

(15) $$\left\{ \begin{array}{l} S_{1}(i^{\prime },j^{\prime })=F_{2}(F_{1}(P_{1}(i,j)))\\ C_{1}(i^{\prime },j^{\prime })=S_{1}(i^{\prime },j^{\prime })⊕K_{1}(i^{\prime },j^{\prime })⊕K_{2}(i^{\prime },j^{\prime }) \end{array}\right.,$$

where *F*_1_ is the first intra-block pixel displacement and *F*_2_ is the second inter-block displacement. Image *S*_1_ represents the permutation-only image after two permutations, *K*_1_ is the diffusion XOR matrix for the first time, and *K*_2_ is the diffusion XOR matrix for the second time. Position (*i*′, *j*′) is the transposition of Position (*i*, *j*).

The encryption process for plain image *P*_2_ is as follows:

(16) $$\left\{ \begin{array}{l} S_{2}(i^{\prime },j^{\prime })=F_{2}(F_{1}(P_{2}(i,j)))\\ C_{2}(i^{\prime },j^{\prime })=S_{2}(i^{\prime },j^{\prime })⊕K_{1}(i^{\prime },j^{\prime })⊕K_{2}(i^{\prime },j^{\prime }) \end{array}\right..$$

According to proposition 2, image *S*_1_ and *S*_2_ after two diffusions can be considered equivalent to those being processed by one linearly transformed diffusion.

(17) $$\left\{ \begin{array}{l} S_{1}(i^{\prime },j^{\prime })=F_{2}(F_{1}(P_{1}(i,j)))\\ C_{1}(i^{\prime },j^{\prime })=S_{1}(i^{\prime },j^{\prime })⊕K(i^{\prime },j^{\prime }) \end{array}\right.,$$

(18) $$\left\{ \begin{array}{l} S_{2}(i^{\prime },j^{\prime })=F_{2}(F_{1}(P_{2}(i,j)))\\ C_{2}(i^{\prime },j^{\prime })=S_{2}(i^{\prime },j^{\prime })⊕K(i^{\prime },j^{\prime }) \end{array}\right.,$$

where *K* (*i*′, *j*′) is the XOR result of *K*_1_ (*i*′, *j*′) and *K*_2_ (*i*′, *j*′).

Since the permutation rules for *P*_1_ and *P*_2_ are the same, the permutation positions for *P*_1_ and *P*_2_ are the same. Now, assuming that the number of different pixel values *n* = 1, there is only one different pixel value at (*i*″, *j*″) between *P*_1_ and *P*_2_. Therefore, in XOR, all pixels at the same positions will have the same values, except the one at (*i*″, *j*″).

(19) $$\left\{ \begin{array}{l} C_{1}(i^{\prime \prime },j^{\prime \prime })=S_{1}(i^{\prime \prime },j^{\prime \prime })⊕K(i^{\prime \prime },j^{\prime \prime })\\ C_{2}(i^{\prime \prime },j^{\prime \prime })=S_{2}(i^{\prime \prime },j^{\prime \prime })⊕K(i^{\prime \prime },j^{\prime \prime }) \end{array}\right..$$

Then,

(20) $$C_{1}(i^{\prime \prime },j^{\prime \prime })\neq C_{2}(i^{\prime \prime },j^{\prime \prime }).$$

Change the pixel values of *P*_1_(1,1) and *P*_1_(1,2) to obtain image *P*′.

(21) $$\{\begin{array}{c} P^{\prime }(1,1)=\mathrm{mod}(P_{1}(1,1)+1256)\\ P^{\prime }(1,2)=\mathrm{mod}(P_{1}(1,2)-1256) \end{array}.$$

Change the pixel values of *P*_1_(1,1) and *P*_1_(1,3) to obtain image *P*″.

(22) $$\{\begin{array}{c} P^{\prime \prime }(1,1)=\mathrm{mod}(P_{1}(1,1)+1256)\\ P^{\prime \prime }(1,3)=\mathrm{mod}(P_{1}(1,3)-1256) \end{array}.$$

The image *P*′ is encrypted to obtain the cipher image *C*_51_, and the image *P*″ is encrypted to obtain the cipher image *C*_52_. There are two different pixels between *C*_1_ and *C*_51_.

(23) $$C_{56}=C_{1}⊕C_{51}.$$

In this case, *C*_56_ has two non-zero values, the positions of the two non-zero values are represented by (*X*_11_, *Y*_11_) and (*X*_12_, *Y*_12_), where *X* represents the row and *Y* represents the column. These two non-zero values are the encrypted positions of *P*′ (1,1) and *P*′ (1,2).

There are two different pixels between *C*_1_ and *C*_52_.

(24) $$C_{57}=C_{1}⊕C_{52}.$$

In this case, *C*_57_ has two non-zero values, the positions of the two non-zero values are represented by (*X*_13_, *Y*_13_) and (*X*_14_, *Y*_14_). These two non-zero values are the encrypted positions of *P*″ (1,1) and *P*″ (1,3).

So, (*X*_11_, *Y*_11_) is the same position as (*X*_13_, *Y*_13_), and (*X*_12_, *Y*_12_) is different from (*X*_14_, *Y*_14_). The comparison between *C*_51_ and *C*_52_ shows that there is only one pixel difference. □

### 3.2. Elimination of Diffusion Effect

#### 3.2.1. Method 1

In the original encryption scheme, a 256 × 256 × 3 plain image **P** was used, which was divided into three channel images: $P_{R}={\left\{P_{R}(i,\;j)\right\}}_{i=1,\;j=1}^{256,256}$, $P_{G}={\left\{P_{G}(i,\;j)\right\}}_{i=1,\;j=1}^{256,256}$ and $P_{B}={\left\{P_{B}(i,\;j)\right\}}_{i=1,\;j=1}^{256,256}$. Three channels are encrypted to obtain $C_{R}={\left\{C_{R}(i,\;j)\right\}}_{i=1,\;j=1}^{256,256}$, $C_{G}={\left\{C_{G}(i,\;j)\right\}}_{i=1,\;j=1}^{256,256}$ and $C_{B}={\left\{C_{B}(i,\;j)\right\}}_{i=1,\;j=1}^{256,256}$. The color cipher image **C** is obtained by merging the three channels. The original encryption scheme is expressed as follows:

(25) $$\left\{ \begin{array}{l} C_{R}(i,j)=F_{2}(F_{1}(P_{R}(i,j)))⊕K_{1}(i,j)⊕K_{2}(i,j)\\ C_{G}(i,j)=F_{2}(F_{1}(P_{G}(i,j)))⊕K_{1}(i,j)⊕K_{3}(i,j)\\ C_{B}(i,j)=F_{2}(F_{1}(P_{B}(i,j)))⊕K_{1}(i,j)⊕K_{4}(i,j) \end{array}\right.,$$

where *F*_1_ is the first intra-block pixel permutation and *F*_2_ is the second inter-block permutation. In the original encryption scheme, the same permutation matrices *F*_1_ and *F*_2_ were used for the three channels. Matrix *K*_1_ is the same matrix used for the first diffusion of the three channels, while *K*_2_, *K*_3_, and *K*_4_ are the same for the second diffusion of the three channels.

Since the original scheme used different diffusion matrices for **P**_R_, **P**_G_ and **P**_B_, it is necessary to consider the matrices used in the two diffusions. According to proposition 2, two diffusion matrices are transformed into one diffusion matrix for XOR operation.

(26) $$\left\{ \begin{array}{l} C_{R}(i,j)=F_{2}(F_{1}(P_{R}(i,j)))⊕K_{12}(i,j)\\ C_{G}(i,j)=F_{2}(F_{1}(P_{G}(i,j)))⊕K_{13}(i,j)\\ C_{B}(i,j)=F_{2}(F_{1}(P_{B}(i,j)))⊕K_{14}(i,j) \end{array}\right.,$$

where *K*_12_ is the XOR matrix of *K*_1_ and *K*_2_, *K*_13_ is the XOR matrix of *K*_1_ and *K*_3_, and *K*_14_ is the XOR matrix of *K*_1_ and *K*_4_.

**Figure 1:**
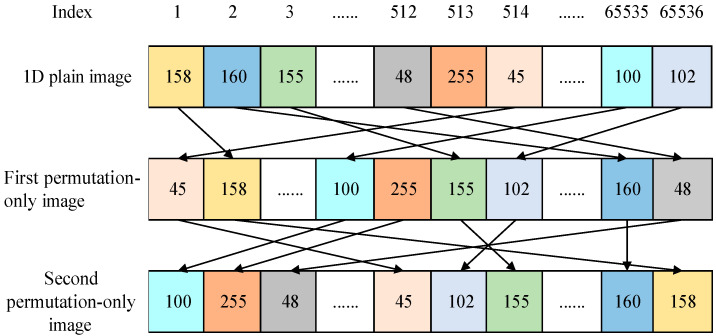
An example of second permutation for a 1D plain image.

As shown in Figure 1, the position of the plain image is still one-to-one mapped to a certain position of the second permutation-only image. So, the two permutation matrices *F*_1_ and *F*_2_ can be linearly transformed into a permutation matrix *F*, as shown in the following formula:

(27) $$\left\{ \begin{array}{l} C_{R}(i,j)=F(P_{R}(i,j))⊕K_{12}(i,j)\\ C_{G}(i,j)=F(P_{G}(i,j))⊕K_{13}(i,j)\\ C_{B}(i,j)=F(P_{B}(i,j))⊕K_{14}(i,j) \end{array}\right..$$

Then, we can construct an all-zero image **P**_0_ of size 256 × 256 × 3 and use the all-zero matrix to obtain the diffusion matrix. The constructed all-zero image **P**_0_ is shown as follows:

(28) $$P_{0}={\left[\begin{array}{ccc} 0 & \cdots & 0\\ \vdots & \ddots & \vdots \\ 0 & \cdots & 0 \end{array}\right]}_{256\times 256\times 3}.$$

We divide the image **P**_0_ into three channels $P_{0}^{R}={\left\{P_{0}^{R}(i,\;j)\right\}}_{i=1,\;j=1}^{256,256}$, $P_{0}^{G}$ = ${\{P_{0}^{G}(i,\;j)\}}_{i=1,\;j=1}^{256,256}$ and $P_{0}^{B}$ = ${\left\{P_{0}^{B}(i,\;j)\right\}}_{i=1,\;j=1}^{256,256}$. After encryption, the cipher images of three channel are $C_{0}^{R}={\left\{C_{0}^{R}(i,\;j)\right\}}_{i=1,\;j=1}^{256,256}$, $C_{0}^{G}={\left\{C_{0}^{G}(i,\;j)\right\}}_{i=1,\;j=1}^{256,256}$ and $C_{0}^{B}={\left\{C_{0}^{B}(i,\;j)\right\}}_{i=1,\;j=1}^{256,256}$. The three channel images $C_{0}^{R}$, $C_{0}^{G}$ and $C_{0}^{B}$ are merged into the color cipher image **C**_0_.

(29) $$\left\{ \begin{array}{l} C_{0}^{R}(i,j)=F(P_{0}^{R}(i,j))⊕K_{12}(i,j)\\ C_{0}^{G}(i,j)=F(P_{0}^{G}(i,j))⊕K_{13}(i,j)\\ C_{0}^{B}(i,j)=F(P_{0}^{B}(i,j))⊕K_{14}(i,j) \end{array}\right..$$

An all-zero matrix is still an all-zero zero matrix after permutation. As follows, Equation (29) can be transformed into Equation (30).

(30) $$\left\{ \begin{array}{l} C_{0}^{R}(i,j)=P_{0}^{R}(i,j)⊕K_{12}(i,j)\\ C_{0}^{G}(i,j)=P_{0}^{G}(i,j)⊕K_{13}(i,j)\\ C_{0}^{B}(i,j)=P_{0}^{B}(i,j)⊕K_{14}(i,j) \end{array}\right..$$

According to proposition 1, three diffusion matrices *K*_12_, *K*_13_ and *K*_14_ can be obtained.

(31) $$\left\{ \begin{array}{l} C_{0}^{R}(i,j)=K_{12}(i,j)\\ C_{0}^{G}(i,j)=K_{13}(i,j)\\ C_{0}^{B}(i,j)=K_{14}(i,j) \end{array}\right..$$

**Figure 2:**
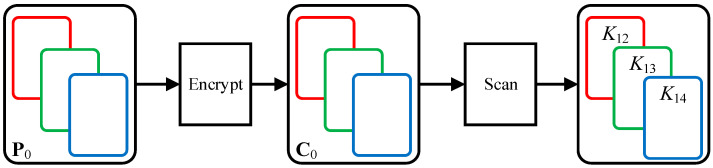
The process of obtaining three diffusion matrices.

The process of obtaining three diffusion matrices is shown in Figure 2. The all-zero image **P**_0_ is encrypted to obtain the merged cipher image **C**_0_. The three channel images $C_{0}^{R}$, $C_{0}^{G}$ and $C_{0}^{B}$ of cipher image **C**_0_ can be transformed into three diffusion matrices *K*_12_, *K*_13_ and *K*_14_.

In method 1, the obtained diffusion matrix is used to eliminate the diffusion effect, and the main steps are described as follows:

**Step 1**  Construct an all-zero image **P**_0_ with 256 × 256 × 3, and the influence of two permutations is eliminated by the all-zero matrix.
**Step 2**  Three equivalent diffusion matrices, *K*_12_, *K*_13_, and *K*_14_, are obtained.
**Step 3**  Then, permutation-only images can be obtained through Equation (32). In the cipher image **C**, the red channel image **C**_R_ XOR diffusion matrix *K*_12_ is permutation-only image **S**_R_, the green channel image **C**_G_ XOR diffusion matrix *K*_13_ is permutation-only image **S**_G_, and the red channel image **C**_B_ XOR diffusion matrix *K*_14_ is permutation-only image **S**_B_. Three channel images, **S**_R_, **S**_G_, and **S**_B_, are merged to obtain color permutation-only image **S**.
  (32) $$\left\{ \begin{array}{l} S_{R}(i,j)=C_{R}(i,j)⊕K_{12}(i,j)\\ S_{G}(i,j)=C_{G}(i,j)⊕K_{13}(i,j)\\ S_{B}(i,j)=C_{B}(i,j)⊕K_{14}(i,j) \end{array}\right..$$

**Figure 3:**
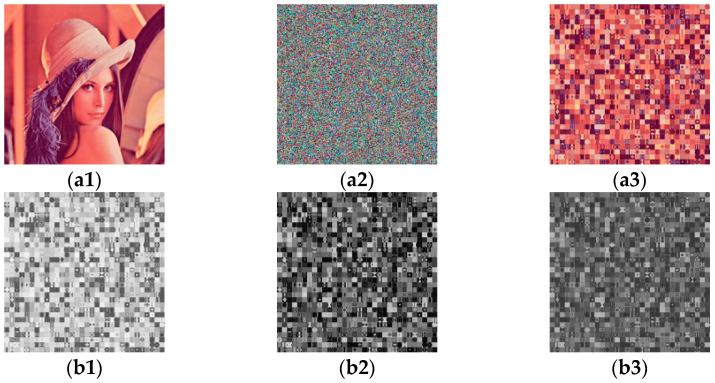
Experiments of method 1: (**a1**) a plain image; (**a2**) the cipher image of (**a1**); (**a3**) permutation-only image of (**a2**); (**b1**–**b3**) permutation-only images from the three channels of (**a2**).

Figure 3 shows the experiments of method 1. Figure 3(a1) is a plain image and Figure 3(a2) is the cipher image of Figure 3(a1). Figure 3(a3) is a permutation-only image of Figure 3(a2). Figure 3(b1–b3) are permutation-only images from the three channels of Figure 3(a2).

#### 3.2.2. Method 2

In method 2, We can construct 256 plain image ${\left\{Px\right\}}_{x=0}^{255}$. The plain images ${\left\{Px\right\}}_{x=0}^{255}$ are encrypted to obtain the cipher images ${\left\{Cx\right\}}_{x=0}^{255}$. The three channel images of the cipher image ${\left\{Cx\right\}}_{x=0}^{255}$ are ${\left\{Crx\right\}}_{x=0}^{255}$, ${\left\{Cgx\right\}}_{x=0}^{255}$ and ${\left\{Cbx\right\}}_{x=0}^{255}$. The plain image ${\left\{Px\right\}}_{x=0}^{255}$ is constructed as shown in Equation (33).

(33) $$P_{x}={\left[\begin{array}{cccc} x & x & \cdots & x\\ x & x & \cdots & x\\ \vdots & \vdots & \ddots & \vdots \\ x & x & x & x \end{array}\right]}_{256\times 256\times 3},$$

where *x* = 0, 1, 2, …, 255. The *x* value in the plain image ${\left\{Px\right\}}_{x=0}^{255}$ changes from 0 to 255.

Thus, 256 different plain images ${\left\{Px\right\}}_{x=0}^{255}$ are encrypted to obtain 256 different cipher images ${\left\{Cx\right\}}_{x=0}^{255}$. If all the (*i*, *j*)th pixels of plain images ${\left\{Px\right\}}_{x=0}^{255}$ are used to form a vector **X** = ${\left\{x\right\}}_{x=0}^{255}$, each of the elements of **X** are different. According to proposition 3, the encrypted cipher image of the plain image always has a unique corresponding position, and each element is mapped one-to-one. So, a given pixel in the target cipher image needs to be decrypted, then the pixel value of the cipher image can be compared with the pixel value at the same position in 256 different cipher images ${\left\{Cx\right\}}_{x=0}^{255}$. For a given cipher image **C**, the three channel images are **C**_R_, **C**_G_, and **C**_B_. Through Equations (34) and (35), the permutation-only images $S_{R}={\left\{S_{R}(i,\;j)\right\}}_{i=1,\;j=1}^{256,256}$, $S_{G}={\left\{S_{G}(i,\;j)\right\}}_{i=1,\;j=1}^{256,256}$ and $S_{B}={\left\{S_{B}(i,\;j)\right\}}_{i=1,\;j=1}^{256,256}$ of three channels are retrieved.

(34) $$\{\begin{array}{c} C_{R}(i,j)=Cr_{x}(i,j)\\ C_{G}(i,j)=Cg_{x}(i,j)\\ C_{B}(i,j)=Cb_{x}(i,j) \end{array},$$

(35) $$\{\begin{array}{c} S_{R}(i,j)=x\\ S_{G}(i,j)=x\\ S_{B}(i,j)=x \end{array}.$$

**Figure 4:**
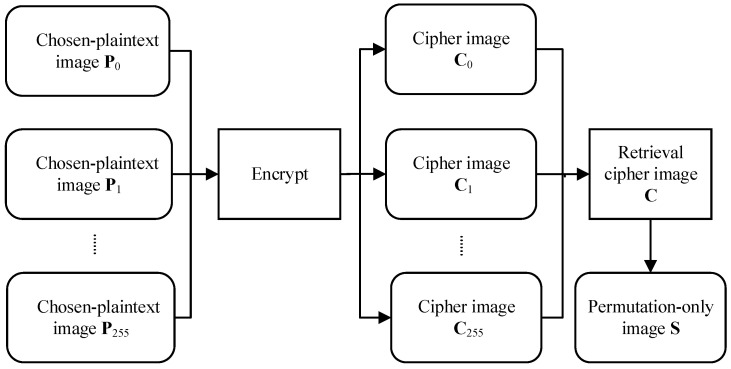
Process of eliminating diffusion effect in method 2.

**Figure 5:**
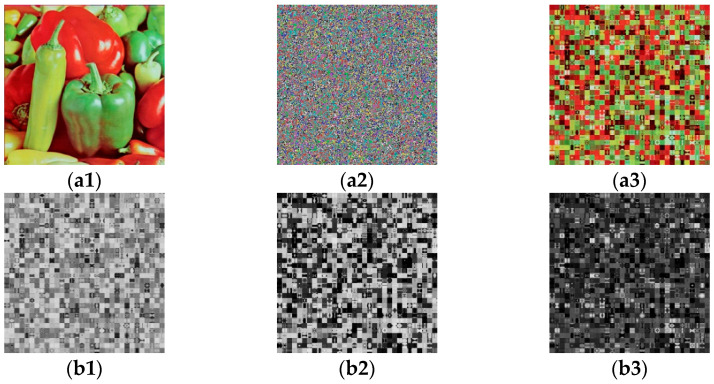
Experiments of method 1: (**a1**) a plain image; (**a2**) the cipher image of (**a1**); (**a3**) permutation-only image of (**a2**); (**b1**–**b3**) are permutation-only images from the three channels of (**a2**).

Figure 4 shows the process of eliminating the diffusion effect, where the color permutation-only image **S** of color cipher image **C** is successfully obtained. Here, **S** is the combined image of three channel images **S**_R_, **S**_G_, and **S**_B_. Figure 5 shows the experiment of method 2, where Figure 5(a1) is a plain image, Figure 5(a2) is the cipher image of Figure 5(a1), Figure 5(a3) is the permutation-only image of Figure 5(a2), and Figure 5(b1–b3) are permutation-only images from the three channels of Figure 5(a2).

### 3.3. Obtain Equivalent Permutation Mapping

In the previous section, the effects of diffusion can be eliminated by means of methods 1 and 2. In this way, we can obtain a permutation-only image of all the cipher images.

In this section, the substitution matrices *F*_1_ and *F*_2_ used in **P**_R_, **P**_G_ and **P**_B_ in the original encryption scheme are the same. Two permutation matrices, *F*_1_ and *F*_2_, can be linearly transformed into one permutation matrix, *F*, so the permutation matrix *F* of each of the three channels is universal.

Cryptanalysis is used in [34] to obtain equivalent permutation matrices on the basis of a permutation-only image. Therefore, we can construct the chosen-plaintext image to obtain the corresponding equivalent permutation mapping **F**′, so that we can eliminate the influence of two permutations and the plain image **P** can be restored.

Based on Lemma 1 in [35], equivalent permutation mapping can be revealed by *n* pairs of plain/cipher images.

(36) $$n\geq \left\lceil \log_{g}(MN)\right\rceil ,$$

where *g* is the gray level of the image and *M* and *N* are the rows and columns of the plain image. In the original encryption scheme, 256 × 256 × 3 plain image is used, which is divided into three 256 × 256 channels for permutation transformation. 

The size of each channel is 256 × 256, which is a total of 65,536 pixel values. Therefore, *g* = 256, *M* = 256, *N* = 256, *n* pairs of plain/cipher images can be obtained from Equation (37).

(37) $$2=\left\lceil \log_{256}(256\times 256)\right\rceil .$$

By calculating *n* = 2, the corresponding equivalent permutation map **F**′ can be solved by using two pairs of plain/cipher images. 

The three channel images **P**_R_, **P**_G_, and **P**_B_ are stretched into column vectors, which can be expanded to a two-digit representation in base 256.

(38) $$A={\left[\begin{array}{ccccc} \left(0)(0\right) & \left(0)(1\right) & \left(0)(2\right) & \cdots & \left(0)(255\right)\\ \left(1)(0\right) & \left(1)(1\right) & \left(1)(2\right) & \cdots & \left(1)(255\right)\\ \vdots & \vdots & \vdots & \ddots & \vdots \\ \left(254)(0\right) & \left(254)(1\right) & \left(254)(2\right) & \cdots & \left(254)(255\right)\\ \left(255)(0\right) & \left(255)(1\right) & \left(255)(2\right) & \cdots & \left(255)(255\right) \end{array}\right]}_{256\times 256\times 3},$$

where the element (*a*1) (*a*2) corresponds to 256 × *a*1 + *a*2 of **A**. The chosen-plaintext image **A** is divided into two bitplane images, **A**_1_ and **A**_2_. Then, **A**_1_ and **A**_2_ are used to construct 2 pairs of plain/cipher images.

(39) $$A_{1}={\left[\begin{array}{ccccc} 0 & 1 & 2 & \cdots & 255\\ 0 & 1 & 2 & \cdots & 255\\ \vdots & \vdots & \vdots & \ddots & \vdots \\ 0 & 1 & 2 & \cdots & 255\\ 0 & 1 & 2 & \cdots & 255 \end{array}\right]}_{256\times 256\times 3},$$

(40) $$A_{2}={\left[\begin{array}{ccccc} 0 & 0 & 0 & \cdots & 0\\ 1 & 1 & 1 & \cdots & 1\\ \vdots & \vdots & \vdots & \ddots & \vdots \\ 254 & 254 & 254 & \cdots & 254\\ 255 & 255 & 255 & \cdots & 255 \end{array}\right]}_{256\times 256\times 3}.$$

Two cipher images **C**_A1_ and **C**_A2_ are obtained by encrypting two plain images, **A**_1_ and **A**_2_. Plain image **A**_1_ is divided into three channel images: $A_{r1}={\left\{A_{r1}(i,\;j)\right\}}_{i=1,\;j=1}^{256,256}$, $A_{g1}={\left\{A_{g1}(i,\;j)\right\}}_{i=1,\;j=1}^{256,256}$ and $A_{b1}={\left\{A_{b1}(i,\;j)\right\}}_{i=1,\;j=1}^{256,256}$. Plain image **A**_2_ is divided into three channel images: $A_{r2}={\left\{A_{r2}(i,\;j)\right\}}_{i=1,\;j=1}^{256,256}$, $A_{g2}={\left\{A_{g2}(i,\;j)\right\}}_{i=1,\;j=1}^{256,256}$ and $A_{b2}={\left\{A_{b2}(i,\;j)\right\}}_{i=1,\;j=1}^{256,256}$. The cipher image **C**_A1_ is divided into three channel images: $C_{r1}={\left\{C_{r1}(i,\;j)\right\}}_{i=1,\;j=1}^{256,256}$, $C_{g1}={\left\{C_{g1}(i,\;j)\right\}}_{i=1,\;j=1}^{256,256}$ and $C_{b1}={\left\{C_{b1}(i,\;j)\right\}}_{i=1,\;j=1}^{256,256}$. The cipher image **C**_A2_ is divided into three channel images: $C_{r2}={\left\{C_{r2}(i,\;j)\right\}}_{i=1,\;j=1}^{256,256}$, $C_{g2}={\left\{C_{g2}(i,\;j)\right\}}_{i=1,\;j=1}^{256,256}$ and $C_{b2}={\left\{C_{b2}(i,\;j)\right\}}_{i=1,\;j=1}^{256,256}$. After the Equation (41) transformation, the red channel images **A***_r_*_1_ and **A***_r_*_2_ are transformed into matrices $P_{r1}={\left\{P_{r1}(i,\;j)\right\}}_{i=1,\;j=1}^{256,256}$. Transforming the green channels **A***_g_*_1_ and **A***_g_*_2_ into matrices $P_{g1}={\left\{P_{g1}(i,\;j)\right\}}_{i=1,\;j=1}^{256,256}$, the blue channel images **A***_b_*_1_ and **A***_b_*_2_ are transformed into a matrix $P_{b1}={\left\{P_{b1}(i,\;j)\right\}}_{i=1,\;j=1}^{256,256}$.

(41) $$\{\begin{array}{c} P_{r1}=256\times A_{r2}+A_{r1}\\ P_{g1}=256\times A_{g2}+A_{g1}\\ P_{b1}=256\times A_{b2}+A_{b1} \end{array}.$$

By method 1 or 2, the permutation-only images $S_{r1}={\left\{S_{r1}(i,\;j)\right\}}_{i=1,\;j=1}^{256,256}$, $S_{g1}={\left\{S_{g1}(i,\;j)\right\}}_{i=1,\;j=1}^{256,256}$ and $S_{b1}={\left\{S_{b1}(i,\;j)\right\}}_{i=1,\;j=1}^{256,256}$ of **C***_r_*_1_, **C***_g_*_1_ and **C***_b_*_1_ are obtained, and permutation-only images $S_{r2}={\left\{S_{r2}(i,\;j)\right\}}_{i=1,\;j=1}^{256,256}$, $S_{g2}={\left\{S_{g2}(i,\;j)\right\}}_{i=1,\;j=1}^{256,256}$ and $S_{b2}={\left\{S_{b2}(i,\;j)\right\}}_{i=1,\;j=1}^{256,256}$ of **C***_r_*_2_, **C***_g_*_2_ and **C***_b_*_2_ are obtained.

(42) $$\{\begin{array}{c} S_{r}=256\times S_{r2}+S_{r1}\\ S_{g}=256\times S_{g2}+S_{g1}\\ S_{b}=256\times S_{b2}+S_{b1} \end{array}.$$

Then, it can be observed that all elements in image **S***_r_* are the permutation order of image **P***_r_*_1_, all elements in image **S***_g_* are the permutation order of image **P***_g_*_1_, and all elements in image **S***_b_* are the permutation order of image **P***_b_*_1_. We know that the permutation matrices of the three channels are the same, and any of the three channels are stretched row by row to form a vector $F^{\prime }={\left\{F^{\prime }(i)\right\}}_{i=1}^{66,256}$. Therefore, **F**′ is an equivalent permutation mapping.

**Figure 6:**
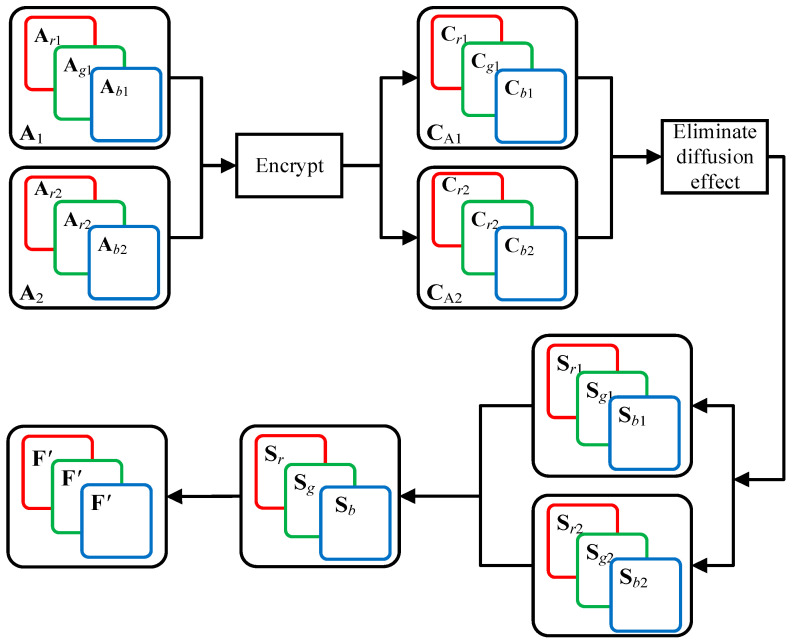
The flow chart to obtain this equivalent permutation mapping.

Figure 6 shows the flow to obtain this equivalent permutation mapping **F**′. The main steps of equivalent permutation mapping are described as follows:

**Step 1**  Two plain images **A**_1_ and **A**_2_ were encrypted to obtain two cipher images: **C**_A1_ and **C**_A2_. The cipher image **C**_A1_ is divided into three channel images: **C***_r_*_1_, **C***_g_*_1_, and **C***_b_*_1_. The cipher image **C**_A2_ is divided into three channel images: **C***_r_*_2_, **C***_g_*_2_, and **C***_b_*_2_.
**Step 2**  By using method 1 or 2, the permutation-only images **S***_r_*_1_, **S***_g_*_1_, and **S***_b_*_1_ of **C***_r_*_1_, **C***_g_*_1_, and **C***_b_*_1_ are obtained first, and after that, the permutation-only images **S***_r_*_2_, **S***_g_*_2_, and **S***_b_*_2_ of **C***_r_*_2_, **C***_g_*_2_, and **C***_b_*_2_ are obtained.

Method 1

**(i)** Three equivalent diffusion matrices, *K*_12_, *K*_13_, and *K*_14_, are known. The red channel image **C***_r_*_1_ is XORed with the diffusion matrix *K*_12_ of the cipher image **C**_A1_ to obtain the permutation-only image **S***_r_*_1_. The green channel image **C***_g_*_1_ is XORed with the diffusion matrix *K*_13_ to obtain the permutation-only image **S***_g_*_1_, and the blue channel image **C***_b_*_1_ is XORed with the diffusion matrix *K*_14_ to obtain the permutation-only image **S***_b_*_1_.

(43) $$\left\{ \begin{array}{l} S_{r1}(i,j)=C_{r1}(i,j)⊕K_{12}(i,j)\\ S_{g1}(i,j)=C_{g1}(i,j)⊕K_{13}(i,j)\\ S_{b1}(i,j)=C_{b1}(i,j)⊕K_{14}(i,j) \end{array}\right..$$

**(ii)** The red channel image **C***_r_*_2_ is XORed with the diffusion matrix *K*_12_ of the cipher image **C**_A2_ to obtain the permutation-only image **S***_r_*_2_. The green channel image **C***_g_*_2_ is XORed with the diffusion matrix *K*_13_ to obtain the permutation-only image **S***_g_*_2_, and the blue channel image **C***_b_*_2_ is XORed with the diffusion matrix *K*_14_ to obtain the permutation-only image **S***_b_*_2_.

(44) $$\left\{ \begin{array}{l} S_{r2}(i,j)=C_{r2}(i,j)⊕K_{12}(i,j)\\ S_{g2}(i,j)=C_{g2}(i,j)⊕K_{13}(i,j)\\ S_{b2}(i,j)=C_{b2}(i,j)⊕K_{14}(i,j) \end{array}\right..$$

Method 2

**(i)** The cipher image **C**_A1_ is divided into three channel images: **C***_r_*_1_, **C***_g_*_1_, and **C***_b_*_1_. The permutation-only images **S***_r_*_1_, **S***_g_*_1_, and **S***_b_*_1_ of the three channel images are retrieved by Equations (45) and (46).

(45) $$\{\begin{array}{c} C_{r1}(i,j)=Cr_{x}(i,j)\\ C_{g1}(i,j)=Cg_{x}(i,j)\\ C_{b1}(i,j)=Cb_{x}(i,j) \end{array},$$

(46) $$\{\begin{array}{c} S_{r1}(i,j)=x\\ S_{g1}(i,j)=x\\ S_{b1}(i,j)=x \end{array}.$$

**(ii)** The cipher image **C**_A2_ is divided into three channel images: **C***_r_*_2_, **C***_g_*_2_ and **C***_b_*_2_. The permutation-only images **S***_r_*_2_, **S***_g_*_2_, and **S***_b_*_2_ of the three channel images are retrieved by Equations (47) and (48).

(47) $$\{\begin{array}{c} C_{r2}(i,j)=Cr_{x}(i,j)\\ C_{g2}(i,j)=Cg_{x}(i,j)\\ C_{b2}(i,j)=Cb_{x}(i,j) \end{array},$$

(48) $$\{\begin{array}{c} S_{r2}(i,j)=x\\ S_{g2}(i,j)=x\\ S_{b2}(i,j)=x \end{array}.$$

**Step 3**  Image **S***_r_*, **S***_g_* and **S***_b_* are obtained from Equation (42). Any of the three channel images are stretched row by row to form $F^{\prime }={\left\{F^{\prime }(i)\right\}}_{i=1}^{66,256}$. Therefore, **F**′ is an equivalent permutation mapping.

**Figure 7:**
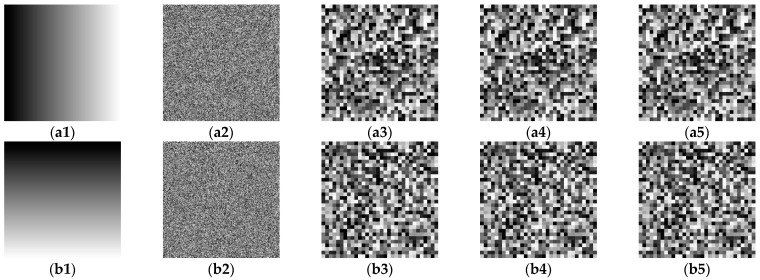
Obtain the permutation-only images of the chosen plain image: the first column (**a1**,**b1**) is the constructed plain images **A**_1_ and **A**_2_; the second column (**a2**,**b2**) is the corresponding cipher images; the third column (**a3**,**b3**), the fourth column (**a4**,**b4**), and the fifth column (**a5**,**b5**) retrieve the second column for three channels of permutation-only images.

Figure 7 shows the permutation-only images of the constructed plain images **A**_1_ and **A**_2_, where the first column is the plain images **A**_1_ and **A**_2_, the second column is the encrypted images of the first column, and the third, fourth, and fifth columns are permutation-only images of the three channels retrieved from the second column. Since the permutation maps of the three channels in the original encryption scheme are the same, the permutation-only images of the three channels are the same. 

### 3.4. Summary of the Attack Strategy

In this section, we provide a detailed overview of the attack process in methods 1 and 2.

#### 3.4.1. Detailed Process of Method 1

**Step 1**  Based on propositions 1 and 2, the three equivalent diffusion matrices *K*_12_, *K*_13_ and *K*_14_ are obtained from the all-zero image (shown in Figure 2) as described in Section 3.2.1. The diffusion effect is eliminated through the known matrices *K*_12_, *K*_13_ and *K*_14_. Then the permutation-only images of the three channel images **C**_R_, **C**_G_ and **C**_B_ are obtained through the three matrices as shown in the sub-steps (shown in Figure 3).
  **Sub-Steps**  The red channel image **C**_R_ XORing equivalent diffusion matrix *K*_12_ is the permutation-only image **S**_R_, the green channel image **C**_G_ XORing diffusion matrix *K*_13_ is the permutation-only image **S**_G_, and the red channel image **C**_B_ XORing diffusion matrix *K*_14_ is the permutation-only image **S**_B_.
**Step 2**  The equivalent permutation mapping **F**′ can be obtained from 2 pairs of plain/cipher images (shown in Figure 6) in Section 3.3. The detailed sub-steps are as follows:
  **Sub-Step 1**   Two plain images **A**_1_ and **A**_2_ were encrypted to obtain two cipher images **C**_A1_ and **C**_A2_. The cipher image **C**_A1_ is divided into three channel images **C***_r_*_1_, **C***_g_*_1_ and **C***_b_*_1_. The cipher image **C**_A2_ is divided into three channel images **C***_r_*_2_, **C***_g_*_2_ and **C***_b_*_2_.
  **Sub-Step 2**   Based on Section 3.2.1, the permutation-only images **S***_r_*_1_, **S***_g_*_1_ and **S***_b_*_1_ of **C***_r_*_1_, **C***_g_*_1_ and **C***_b_*_1_ were obtained, and the permutation-only images **S***_r_*_2_, **S***_g_*_2_ and **S***_b_*_2_ of **C***_r_*_2_, **C***_g_*_2_ and **C***_b_*_2_ were obtained (shown in Figure 7).
  **Sub-Step 3**   **S***_r_*, **S***_g_* and **S***_b_* are obtained from Equation (42). All the three channels are stretched row by row to form an equivalent permutation mapping **F**′.
**Step 3**  Invoke Algorithm 1 to obtain deciphered images **P**_R_, **P**_G_ and **P**_B_. Merge the three channel images for obtaining image **P**.

**Algorithm 1** Obtain the decrypted image **P**.Decryptimg **P** = De_Img(**S***_r_*, **S***_G_*, **S***_b_*, **F**′)1: *w* = 0;2: for *i* from 1 to 2563:   for *j* from 1 to 2564:   [*r*1, *c*1] ← find(**F**′ = *w*); 5:   *d*_*r*(*i*, *j*) ← **S***_r_*(*r*1, *c*1);6:   *d*_*g*(*i*, *j*) ← **S***_g_*(*r*1, *c*1);7:   *d*_*b*(*i*, *j*) ← **S***_b_*(*r*1, *c*1);8:   *w* + 1 ← *w*;9:  end10: end11: **P** ← *d*_*r*, *d*_*g*, *d*_*b* //Merge three channels

#### 3.4.2. Detailed Process of Method 2

**Step 1**  Based on proposition 3, one can construct 256 different plain images ${\left\{P_{x}\right\}}_{x=0}^{255}$ and encrypt them to obtain 256 different cipher images ${\left\{C_{x}\right\}}_{x=0}^{255}$ (shown in Figure 4) in Section 3.2.2. For the three channel images **C**_R_, **C**_G_ and **C**_B_ of the given cipher image **C**. Equations (34) and (35) are used to eliminate the diffusion effect. The permutation-only images **S**_R_, **S**_G_ and **S**_B_ of the three channels are retrieved (shown in Figure 5).
**Step 2**   The equivalent permutation mapping **F**′ can be obtained from 2 pairs of plain/cipher images (shown in Figure 6) as described in Section 3.3. The detailed sub-steps are the same as the three sub-steps of step 2 in Section 3.4.1.
**Step 3**   Invoke Algorithm 1 to obtain deciphered images **P**_R_, **P**_G_ and **P**_B_. Then, image **P** is obtained by merging the three channel images.

### 3.5. Simulation Experiment

#### 3.5.1. Computational Complexity Analysis

In our cryptanalysis, method 1 used three chosen plain images to attack the original encryption scheme, one all-zero image to obtain the diffusion matrix and two chosen plain images to obtain the equivalent permutation mapping. For a cipher image with a size of 256 × 256 × 3, the data complexity is *O* (3 × 256 × 256 × 3), which is close to *O* (2^23^). Method 2 used 258 chosen plain images to attack the original encryption scheme. A total of 256 chosen plain images were used to eliminate the diffusion effect, and 2 chosen plain images were used to obtain the equivalent permutation mapping. Similarly, for a cipher image with a size of 256 × 256 × 3, the data complexity is *O* (258 × 256 × 256 × 3), which is close to *O* (2^26^). More remarkably, method 1 is faster, but method 2 can break the diffusion phase of Majid’s encryption scheme more subtly.

#### 3.5.2. Experimental Results

The simulation experiment was performed on a personal computer (Intel Core i5-10210U 2.11 GHz CPU, 16 GB). All experiments were carried out using MATLAB R2020a. In the simulation experiments, we used the same initial values as the Majid’s scheme. The two attack methods are identical in the steps of obtaining equivalent permutation mapping **F**′ but different in the steps of eliminating diffusion effect. We implemented these two attack methods using some different experimental images for accuracy. It can be found from Table 1 that the two methods will cause time differences due to different computational complexity, and different images will also cause time differences in different attack processes. However, the two solutions we proposed are sufficient in terms of real-time requirements. 

**Figure 8:**
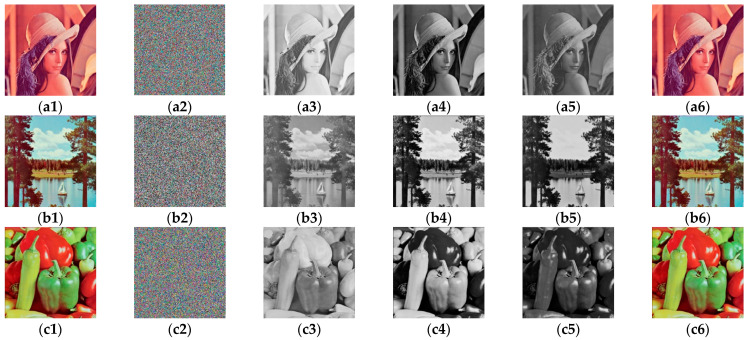
Experimental results of method 1 and method 2: (**a1**,**b1**,**c1**) are plain images; (**a2**,**b2**,**c2**) are the cipher images of (**a1**,**b1**,**c1**); three channel images (**a3**–**a5**) are recovered from (**a2**); three channel images (**b3**–**b5**) are recovered from (**b2**); three channel images (**c3**–**c5**) are recovered from (**c2**); (**a6**,**b6**,**c6**) final deciphered image.

The simulation results of the two attack methods in this paper are shown in Figure 8. Figure 8(a1,b1,c1) are the images of “Lena”, “Sailboat”, and “Pepper”. They are encrypted by the original cryptosystem as plain images, and the encrypted images are shown in Figure 8(a2,b2,c2). Then, we can execute two attack methods, respectively. The encrypted image can be restored to permutation-only images first, and then the decrypted image, as shown in column 3, column 4, and column 5 in Figure 8. Figure 8(a6,b6,c6) show the combined images of the three channels, that is, the recovered decrypted images. After comparison, we find that the decrypted image is consistent with the plain image, which confirms the feasibility of our attack methods.

## 4. Conclusions

In this paper, we cryptanalyzed a new chaotic image encryption technique based on multiple discrete dynamic maps, which adopts one-round encryption and permutation–diffusion structure. (1) The permutation–diffusion structure of one-round execution is found to be insecure because the cipher image generated by the all-zero image is the same as the diffusion matrix of the XOR phase. (2) The original encryption scheme’s secret key is irrelevant to the plain image, leading to the successful attack of the two proposed methods. The simulation results show that both of our methods are feasible.

In view of the original encryption scheme, we put forward some reasonable suggestions. (1) There should be strong correlation between the key and the plain image. (2) Multiple rounds of encryption should be adopted since it is difficult to avoid security problems with only one round of encryption. (3) Add nonlinear substitution phase to the encryption scheme as appropriate. (4) For the diffusion stage, some pixels in the plain image should be fused to complicate the diffusion, so as to avoid obtaining the diffusion matrix directly.

## Figures and Tables

**Table 1:** Execution time (seconds).

Image	Image Size	Encryption	Deciphering	
			Method 1	Method 2
Lena	256 × 256 × 3	1.191755	9.057486	235.661488
Pepper	256 × 256 × 3	1.083255	8.445166	233.262743
Sailboat	256 × 256 × 3	1.058173	8.273460	233.330141

## Data Availability

All results and data obtained can be found in open access publications.

## References

[1] Zhang E., Li M., Yiu S., Du J., Zhu J., Jin G. (2021). Fair hierarchical secret sharing scheme based on smart contract. Inf. Sci..

[2] Zhang E., Li H., Huang Y., Hong S., Zhao L., Ji C. (2022). Practical multi-party private collaborative k-means clustering. Neurocomputing.

[3] Daudigny R., Ledig H., Muller F., Valette F. (2005). SCARE of the DES—(Side channel analysis for reverse engineering of the data encryption standard). Appl. Cryptogr. Netw. Secur. Proc..

[4] Ap W.S., Phan R.C.W., Goi B.M. (2016). Cryptanalysis of a highdefnition image encryption based on AES modifcation. Wirel. Pers. Commun..

[5] Hua Z.Y., Zhou Y.C., Pun C.M., Chen C.L.P. (2015). 2D Sine Logistic modulation map for image encryption. Inf. Sci..

[6] Ua Z.Y., Zhou Y.C. (2016). Image encryption using 2D logisticadjusted-sine map. Inf. Sci..

[7] Enayatifar R., Abdullah A.H., Isnin I.F., Altameem A., Lee M. (2017). Image encryption using a synchronous permutation-difusion technique. Opt. Lasers Eng..

[8] Irfan Y., Majid K. (2018). A New Efficient Digital Image Encryption Based on Inverse Left Almost Semi Group and Lorenz Chaotic System. Entropy.

[9] Diab H., El-Semary A.M. (2018). Secure image cryptosystem with unique key streams via hyper-chaotic system. Signal Process..

[10] Chen J.X., Zhu Z.L., Zhang L.B., Zhang Y.S., Yang B.Q. (2018). Exploiting self-adaptive permutation-difusion and DNA random encoding for secure and efcient image encryption. Signal Process..

[11] Zou C., Wang X., Li H. (2021). Image encryption algorithm with matrix semi-tensor product. Nonlinear Dyn..

[12] Xingyuan W., Maochang Z. (2021). An image encryption algorithm based on hyperchaotic system and DNA coding. Opt. Laser Technol..

[13] Fridrich J. (1997). Image encryption based on chaotic maps. IEEE Int. Conf. Syst..

[14] Zhou Y.C., Bao L., Chen C.L.P. (2014). A new 1D chaotic system for image encryption. Signal Process..

[15] Pak C., Huang L. (2017). A new color image encryption using combination of the 1D chaotic map. Signal Process..

[16] Hua Z., Zhou Y. (2017). Design of image cipher using block-based scrambling and image filtering. Inf. Sci..

[17] Niyat Yaghouti A., Moattar M.H., Torshiz Niazi M. (2017). Color image encryption based on hybrid hyper-chaotic system and cellular automata. Opt. Lasers Eng..

[18] Wu J., Liao X., Yang B. (2018). Image encryption using 2D Hénon-Sine map and DNA approach. Signal Process..

[19] Li M., Wang P.C., Liu Y.F., Fan H.J. (2019). Cryptanalysis of a novel bit-level color image encryption using improved 1D chaotic map. IEEE Access..

[20] Wang H., Xiao D., Chen X., Huang H. (2018). Cryptanalysis and enhancements of image encryption using combination of the 1D chaotic map. Signal Process..

[21] Feng Y., Xinhui G., Hanpeng L., Shihong W. (2021). Differential cryptanalysis of image cipher using block-based scrambling and image filtering. Inf. Sci..

[22] Li M., Lu D.D., Wen W., Ren H., Zhang Y. (2018). Cryptanalyzing a color image encryption scheme based on hybrid hyper-chaotic system and cellular automata. IEEE Access.

[23] Chen J., Chen L., Zhou Y. (2020). Cryptanalysis of a DNA-based image encryption scheme. Inf. Sci..

[24] Hu G.Q., Xiao D., Wang Y., Li X. (2017). Cryptanalysis of a chaotic image cipher using Latin square-based confusion and diffusion. Nonlinear Dyn..

[25] Li M., Lu D.D., Xiang Y., Zhang Y., Ren H. (2019). Cryptanalysis and improvement of a chaotic image cipher using two-round permutation and diffusion. Nonlinear Dyn..

[26] Hsiao H.I., Lee J. (2015). Color image encryption using chaotic nonlinear adaptive filter. Signal Process..

[27] Fan H., Li M., Liu D., Zhang E. (2018). Cryptanalysis of a colour image encryption using chaotic APFM nonlinear adaptive filter. Signal Process..

[28] Sheela S.J., Suresh K.V., Tandur D. (2018). Image encryption based on modified Henon map using hybrid chaotic shift transform. Multimed. Tools Appl..

[29] Zhou K., Xu M., Luo J., Fan H., Li M. (2019). Cryptanalyzing an image encryption based on a modified Henon map using hybrid chaotic shift transform. Digit. Signal Process..

[30] Mondal B., Behera P.K., Gangopadhyay S. (2020). A secure image encryption scheme based on a novel 2D sine-cosine cross-chaotic (SC3) map. J. Real-Time Image Process..

[31] Li M., Wang P., Yue Y., Liu Y. (2021). Cryptanalysis of a secure image encryption scheme based on a novel 2D sine–cosine cross-chaotic map. J. Real-Time Image Process..

[32] Khan M., Masood F. (2019). A novel chaotic image encryption technique based on multiple discrete dynamical maps. Multimed. Tools Appl..

[33] Arroyo D., Diaz J., Rodriguez F.B. (2012). Cryptanalysis of a one round chaos-based substitution permutation network. Signal Process..

[34] Zhang L.Y., Liu Y., Pareschi F., Zhang Y., Wong K.W., Rovatti R., Setti G. (2017). On the security of a class of diffusion mechanisms for image encryption. IEEE Trans. Cybern..

[35] Jolfaei A., Wu X., Muthukkumarasamy V. (2016). On the security of permutation-only image encryption schemes. IEEE Trans. Inf. Forensics Secur..

